# Proteomic and phosphoproteomic measurements enhance ability to predict ex vivo drug response in AML

**DOI:** 10.1186/s12014-022-09367-9

**Published:** 2022-07-27

**Authors:** Sara J. C. Gosline, Cristina Tognon, Michael Nestor, Sunil Joshi, Rucha Modak, Alisa Damnernsawad, Camilo Posso, Jamie Moon, Joshua R. Hansen, Chelsea Hutchinson-Bunch, James C. Pino, Marina A. Gritsenko, Karl K. Weitz, Elie Traer, Jeffrey Tyner, Brian Druker, Anupriya Agarwal, Paul Piehowski, Jason E. McDermott, Karin Rodland

**Affiliations:** 1 Pacific Northwest National Laboratory, Seattle, WA USA; 2 Knight Cancer Institute, Oregon Health & Science University, Portland, OR USA; 3 Division of Hematology & Medical Oncology, Department of Medicine, Oregon Health & Science University, Portland, OR USA; 4 Department of Biology, Faculty of Science, Mahidol University, Bangkok, Thailand; 5 Division of Oncological Sciences, Oregon Health & Science University, Portland, OR USA; 6 Department of Cell, Developmental, and Cancer Biology, Oregon Health & Science University, Portland, OR USA; 7 Department of Molecular Microbiology and Immunology, Oregon Health & Science University, Portland, OR USA

## Abstract

Acute Myeloid Leukemia (AML) affects 20,000 patients in the US annually with a five-year survival rate of approximately 25%. One reason for the low survival rate is the high prevalence of clonal evolution that gives rise to heterogeneous sub-populations of leukemic cells with diverse mutation spectra, which eventually leads to disease relapse. This genetic heterogeneity drives the activation of complex signaling pathways that is reflected at the protein level. This diversity makes it difficult to treat AML with targeted therapy, requiring custom patient treatment protocols tailored to each individual’s leukemia. Toward this end, the Beat AML research program prospectively collected genomic and transcriptomic data from over 1000 AML patients and carried out ex vivo drug sensitivity assays to identify genomic signatures that could predict patient-specific drug responses. However, there are inherent weaknesses in using only genetic and transcriptomic measurements as surrogates of drug response, particularly the absence of direct information about phosphorylation-mediated signal transduction. As a member of the Clinical Proteomic Tumor Analysis Consortium, we have extended the molecular characterization of this cohort by collecting proteomic and phosphoproteomic measurements from a subset of these patient samples (38 in total) to evaluate the hypothesis that proteomic signatures can improve the ability to predict response to 26 drugs in AML ex vivo samples. In this work we describe our systematic, multi-omic approach to evaluate proteomic signatures of drug response and compare protein levels to other markers of drug response such as mutational patterns. We explore the nuances of this approach using two drugs that target key pathways activated in AML: quizartinib (FLT3) and trametinib (Ras/MEK), and show how patient-derived signatures can be interpreted biologically and validated in cell lines. In conclusion, this pilot study demonstrates strong promise for proteomics-based patient stratification to assess drug sensitivity in AML.

**Supplementary Information:**

The online version contains supplementary material available at 10.1186/s12014-022-09367-9.

## Background

Acute myeloid leukemia (AML) is characterized by the incomplete maturation of myeloblasts and their expansion in blood and bone marrow, which impacts healthy blood cell formation resulting in decreased numbers of granulocytes, platelets, and red blood cells [1]. Though the number of FDA-approved treatments for AML has increased significantly over the past five years, prognosis remains poor with a 5-year survival rate of 25% for individuals over the age of 20 [2]. Targeted agents have shown promise in mutationally defined subsets of patients, but due to the genetic evolution of this highly heterogenous disease, drug response is often lost and patients relapse. Proper selection of personalized drugs and drug combinations over the course of a patient’s disease will be required to provide more durable clinical responses, and will require a comprehensive mechanistic evaluation of each patient’s leukemia.

The goal of the Beat AML program was to improve drug selection by collecting large quantities of molecular data together with ex vivo small molecule inhibitor assays performed on freshly isolated patient leukemia cells. In these studies, peripheral blood and bone marrow mononuclear cells from AML patients are isolated and exposed to a panel of approximately 145 drugs over a three-day period and cell viability is used as the primary readout for drug efficacy. Patient genomics and transcriptomics, as well as extensive clinical annotation, enable the stratification of patients by these measures which are more effective than predictions of drug response based on genetics alone [3]. This functional genomic and transcriptomic dataset uncovered numerous novel genetic, transcriptomic, and microenvironmental drivers of AML pathogenesis and drug resistance [4–8].

Proteomic measurements, including measurements of global protein levels and specific phosphosites, have been shown to better identify clinically relevant patterns in patient tumors compared to transcriptomics or genetics alone [9]. This has motivated significant investment by the National Cancer Institute through the Clinical Proteomic Tumor Analysis Consortium (CPTAC) in which patient-derived samples have been assayed using state-of-the-art mass spectrometry (MS) pipelines to produce proteomic and phosphoproteomic measurements of hundreds of tumors in breast, ovary, kidney, head and neck, endometrium, brain and other tissues [10–15]. In each study, these proteomic measurements reveal patterns that are not evident at the genomic or transcriptomic level [9]. Most efforts to study how proteomics signatures can predict drug response have been previously evaluated in cell lines [16, 17] and AML patient samples [18]. More recent efforts have characterized proteomics in patient samples using reverse phase proteomic assays (RPPA) in a pediatric AML cohort [19] as well as focusing explicity on phosphoproteomics measurements in AML related to FLT3 activity [20], showing how measuring protein and phosphorylation activity can better stratify patients and predict drug response. To date, however the integration of proteomic, phosphoproteomic, transcriptomic, and genomic data with drug response has not been evaluated in AML patient samples.

There exist numerous computational modeling and machine learning approaches to predict the response of cancer cell lines to drug perturbation using baseline genomics or transcriptomics [21, 22]. These approaches have been widely successful using genomic data together with subsequent dose response measurements to identify specific signatures capable of predicting which drugs affect cell lines from basal genomic and transcriptomic data of those same cell lines [23, 24]. These datasets have been further supplemented by global proteomic analysis of the same cell line library [25] that have also been used to predict drug response. However, cell line-derived computational models have their flaws, as they sample a limited subset of patient genetics and have been shown to correlate poorly with patient-derived xenograft data of the same tumor type [26], suggesting they are not accurate models of in vivo tumors. There are still ongoing innovations in the computational space that predict drug response from the underlying genomic phenotype [27] including Bayesian approaches [28], variational auto-encoders [29], and deep learning [30]. However, proteomic measurements in cell lines have been shown to provide improvement over drug prediction modeling in numerous cases, suggesting that additional data can improve modeling [16, 31–33]. To date, however, most predictive models are based on cancer cell lines, which are limited in their ability to recapitulate the diversity of genetic backgrounds found in patients and lack potential contributions from the tumor microenvironment.

In this work, we combine the rigorous pre-clinical drug testing and genomic profiling of the Beat AML dataset with patient-derived proteomic and phosphoproteomic measurements to determine the potential for protein-level data to produce robust molecular biomarkers of drug response. Using a small pilot proteomic dataset of 38 patients, we focus on two drugs that target the FLT3 and Ras/MEK pathways in AML (quizartinib and trametinib respectively) and evaluate how the genes, transcripts and proteins measured in each patient sample correlate with drug sensitivity. We expand our analysis to 24 additional drugs to determine how well baseline proteomic and phosphoproteomic measurements can predict drug response compared to genomic or transcriptomic measurements. We then explore the signatures that result from our analysis to determine how best to interpret these results biologically, by both evaluating their role in signaling networks and also assessing their expression in drug-resistant cell lines. Together this work represents a robust toolkit by which protein-derived signatures can be used to predict drug response and understand the biological pathways these signatures represent.

## Methods

### Experimental design

Our overall experimental design is depicted in Additional file 2: Figure S1. It entails subjecting patient AML samples to genomic and proteomic analysis and ex-vivo drug screening followed by the construction of predictive models of drug response for each type of data collected. We then use the signatures determined by the model to assess their performance in cross-validation experiments, explore their role in biological networks, and then validlate them in cell lines.The data collected are summarized in Additional file 1: Table S1.

### Sample collection

Samples were collected and processed as described in detail previously [3]. Briefly, all patients gave informed consent to participate in the Beat AML study, which had the approval and guidance of the Institutional Review Boards (IRB) from participating institutions. All samples used in this manuscript were collected at Oregon Health & Science University with a broad ‘research use’ clause. Mononuclear cells (MNCs) were isolated from freshly obtained bone marrow or peripheral blood samples from AML patients via Ficoll gradient centrifugation. Isolated MNCs were utilized for genomic (500 × WES; RNA-seq) and ex vivo functional drug screens. WES and RNA-seq were performed using standard methods and data analysis was performed as previously described [3]. Clinical, prognostic, genetic, cytogenetic and pathologic laboratory values as well as treatment and outcome data were manually curated from the patient electronic medical records (EMR). Patients were assigned a specific diagnosis based on the prioritization of genetic and clinical factors as determined by WHO guidelines. We selected 38 unique patients from our ongoing study that had complete proteomic and phosphoproteomic measurements.

### Ex vivo drug screening analysis

For drug sensitivity assays, 10,000 viable cells were dispensed into each well of a 384-well plate containing 7 point, threefold dilution, drug concentration series from a library of small molecule inhibitors. Cells were incubated with the drugs in RPMI media containing 10% FBS without supplementary cytokines. After 3 days of culture at 37 °C in 5% CO_2_, MTS reagent (CellTiter96 AQueous One; Promega) was added, the optical density was measured at 490 nm, and raw absorbance values were adjusted to a reference blank value and then used to determine cell viability (normalized to untreated control wells). Ex vivo functional drug screen data processing was performed as described, and dose response curve-fitting was carried out using the probit regression on quality-controlled data as in our previous work [3].

### Protein digestion and tandem mass tag (TMT) labeling

Sample preparation for proteomics was based on the protocol developed under the CPTAC consortium with minimal modifications [34]. Patient cell pellets were lysed with 500 µL fresh lysis buffer, containing 8 M urea (Sigma-Aldrich), 50 mM Tris pH 8.0, 75 mM sodium chloride, 1 mM ethylenediamine tetra-acetic acid, 2 µg/mL Aprotinin (Sigma-Aldrich), 10 mg/mL Leupeptin (Roche), 1 mM PMSF in EtOH, 10 mM sodium fluoride, 1% of phosphatase inhibitor cocktail 2 and 3 (Sigma-Aldrich), 20 µM PUGNAc, and 0.01 U/µ/µL Benzonase. The samples were then vortexed for 10 s and placed in a thermomixer for 15 min at 4 °C and 800 RPM. Vortexing was repeated and the samples incubated again for 15 min utilizing the same settings. After incubation, the samples were centrifuged for 10 min at 4 °C and 18,000 rcf to remove cell debris. The supernatant was then transferred to a fresh tube. A BCA (ThermoFisher) assay was performed on the supernatant to determine protein yield.

Protein concentrations were normalized to the same concentration prior to beginning digestion. The sample was reduced with 5 mM dithiothreitol (DTT) (Sigma-Aldrich) for 1 h at 37 °C and 800 rpm. Reduced cystines were alkylated with 10 mM iodacetamide (IAA) (Sigma-Aldrich) for 45 min at 25 °C and 800 rpm in the dark. The sample was diluted fourfold with 50 mM Tris HCL pH 8.0 and then Lys-C (Wako) was added at a 1:20 enzyme:substrate ratio, followed by incubation for 2 h at 25 °C, shaking at 800 rpm. Trypsin (Promega) was then added at a 1:20 enzyme:substrate ratio, followed by a 14-h incubation at 25 °C and 800 rpm. The sample was quenched by adding formic acid to 1% by volume, and centrifuged for 15 min at 1500 rcf to remove any remaining cell debris. Peptides samples were desalted using a C18 solid phase extraction (SPE) cartridge (Waters Sep-Pak).

After drying down SPE eluates, each sample was reconstituted with 50 mM HEPES, pH 8.5 to a concentration of 5 µ/µ/µL. Each isobaric tag aliquot was dissolved in 250 µL anhydrous acetonitrile to a final concentration of 20 µg/µ/µL. The tag was added to the sample at a 1:1 peptide:label ratio and incubated for 1 h at 25 °C and 400 rpm and then diluted to 2.5 mg/mL with 50 mM HEPES pH 8.5, 20% acetonitrile (ACN). Finally, the reaction was quenched with 5% hydroxylamine and incubated for 15 min at 25 °C and 400 rpm. The samples were then combined per each plex set and concentrated in a speed-vac before a final C18 SPE cleanup. Each 11-plex experiment was fractionated into 96 fractions by high pH reversed phase separation, followed by concatenation into 24 or 12 global fractions for MS analysis.

### Phosphopeptide enrichment using IMAC

The global samples were further concatenated to create 6 samples per plex for further enrichment. Fe^3+^-NTA-agarose beads were freshly prepared using Ni–NTA-agarose beads (Qiagen). Sample peptides were reconstituted to a 0.5 µg/µL concentration with 80% ACN, 0.1% TFA and incubated with 40 mL of the bead suspension for 30 min at RT in a thermomixer set at 800 rpm. After incubation the beads were washed with 100 mL 80% ACN, 0.1% TFA and 50 mL 1% FA to remove any non-specific binding. Phosphopetides were eluted off beads with 210 mL 500 mM K_2_HPO_4_, pH 7.0 directly onto C18 stage tips and eluted from C18 material with 60 mL 50% ACN, 0.1% FA. Samples were dried in speed-vac concentrator for storage and reconstituted with 12 mL 3% ACN, 0.1% FA immediately prior to MS analysis.

### LC–MS/MS analysis

Proteomic fractions were separated using a Waters nano-Aquity UPLC system (Waters) equipped with a 75 um I.D. × 25 cm length C18 column packed in-house with 1.9 um ReproSil-Pur 120 C18-AQ (Dr. Maisch GmbH). A 120-min gradient of 95% mobile phase A (0.1% (v/v) formic acid in water) to 19% mobile phase B (0.1% (v/v) FA in acetonitrile) was applied to each fraction. The separation was coupled to either a Thermo Orbitrap™ Fusion Lumos™ (patient samples) or Q Exactive™ HF (cell lines) Hybrid Quadrupole-Orbitrap™ mass spectrometer for MS/MS analysis. MS Spectra were collected from 350 to 1800 m/z at a mass resolution setting of 60,000. A top speed method was used for the collection of MS2 spectra at a mass resolution of 50 K. An isolation window of 0.7 m/z was used for higher energy collision dissociation (HCD), singly charged species were excluded, and the dynamic exclusion window was 45 s. For the Fusion Lumos™, a top speed method was used for the collection of MS2 spectra at a mass resolution of 50 K. For the Q Exactive™ HF experiments, a top 16 method was used for the collection of MS [2] spectra at a mass resolution of 30 K.

### TMT global proteomics data processing

All Thermo RAW files were processed using mzRefinery to correct for mass calibration errors, and then spectra were searched with MS-GF + v9881 [35–37] to match against the human reference protein sequence database downloaded in April of 2018 (71,599 proteins), combined with common contaminants (e.g., trypsin, keratin). A partially tryptic search was used with a ± 10 parts per million (ppm) parent ion mass tolerance. A reversed sequence decoy database approach was used for false discovery rate calculation. MS-GF + considered static carbamidomethylation (+ 57.0215 Da) on Cys residues and TMT modification (+ 229.1629 Da) on the peptide N terminus and Lys residues, and dynamic oxidation (+ 15.9949 Da) on Met residues. The resulting peptide identifications were filtered to a 1% false discovery rate at the unique peptide level. A sequence coverage minimum of 6 per 1000 amino acids was used to maintain a 1% FDR at the protein level after assembly by parsimonious inference.

The intensities of TMT 11 reporter ions were extracted using MASIC software [38]. Extracted intensities were then linked to peptide-to-spectrum matches (PSMs) passing the FDR thresholds described above. Relative protein abundance was calculated as the ratio of sample abundance to reference channel abundance, using the summed reporter ion intensities from peptides that could be uniquely mapped to a gene. The relative abundances were log2 transformed and zero-centered for each gene to obtain final relative abundance values. We identified 8963 distinct proteins across 38 patients, with some variability as depicted in Additional file 2: Figure S2A.

### TMT phosphoproteomics data processing

IMAC enriched fraction datasets were searched as described above with the addition of a dynamic phosphorylation (+ 79.9663 Da) modification on Ser, Thr, or Tyr residues. The phosphoproteomic data were further processed with the Ascore algorithm [39] for phosphorylation site localization, and the top-scoring assignments were reported. To account for sample loading biases in the phosphoproteome analysis, we applied the same correction factors derived from median-centering of the global proteomic dataset for normalization. We identified 45,806 distinct phosphopeptides across 38 patients, that mapped to 31,788 pSer sites, 7395 pT sites, and 1259 pTyr sites. Additional file 2: Figure S2B shows the relationship of these sites to their annotation in databases of known kinase-substrate interactions.

All proteomic data can be found on our synapse site (http://synapse.org/ptrc). The cohort is spread across three tranches, as described in Table 1 below.

**Table 1:** Location of processed proteomics files on Synapse

Patients	Data type	File	Table
Primary patient cohort	Proteomics	syn22130778	syn22172602
Patients with Sorafenib treatment	Proteomics	syn22313435	syn22314121
Patients with drug combination	Proteomics	syn25672089	syn22156810
Primary patient cohort	Phosphoproteomics	syn24610481	syn24227903
Patients with Sorafenib treatment	Phosphoproteomics	syn24227680	syn24228075
Patients with drug combination	Phosphoproteomics	syn24240156	syn24240355

### Identifying drugs and samples for analysis

The list of available data for each patient is in Additional file 1: Table S1. Although ~ 145 total compounds were tested in the drug panels, we filtered the drugs in this study to collect those that exhibited a range of responses across the 38 patients as determined by area under the curve (AUC) of the dose response. AUC correlates to the amount of drug required to reduce cell viability, so higher AUC values mean the samples are less sensitive to the drug, and lower AUC values indicate the samples are more sensitive. We selected drugs for which at least 10% or 2 (whichever was greater) samples exhibited an AUC less than 100 (determined to be sensitive in previous work [3]). This selection produced a “balanced” distribution of AUC scores to enable our downstream analysis. We also added Gilteritinib (ASP-2215) to the panel as it is currently being evaluated in numerous clinical trials. Drug responses varied across the 38 patients for 26 drugs, with AUC values ranging from 14.7 to 186.3. Despite some missingness in the data, we were able to use these values to compare the efficacy of genomics, transcriptomics, proteomics, and phosphoproteomics to model drug sensitivity based on the available data.

### Linear models of proteomics and drug response

We constructed linear models for each of the 26 different drugs across up to 38 patients (depending on how many patient samples were evaluated with that drug) by regressing the AUC values (which ranged between 0 and ~ 300, as depicted in Fig. 2A) against the molecular data shown in Additional file 1: Table S1 and Additional file 2: Figure S2A. The input data for each model were each scaled slightly differently: the genetic mutations were represented as a binary matrix in which a 1 represented the presence of a somatic mutation and a 0 represented no mutation, the transcriptomics was represented by Counts per Million (CPM) of gene expression values, while proteomics and phosphoproteomics were represented as the log ratio of gene/phosphosites compared to the reference sample described above.

For each combination of drug and data type, we constructed a linear model Y ~ X where Y represents the vector of AUC values and X represents the molecular measurements for that patient. We used three different linear modeling approaches to reduce the number of features selected by the model: LASSO regression [40], Elastic Net Regression [41], and logistic regression as implemented by the `glmnet` package [42]. For the logistic regression, we discretized the AUC by representing Y as a binary variable, where 1 represented an AUC greater than 100 (patient is resistant to drug) and 0 if the AUC is less than 100 (patient is sensitive to drug).

For each model, we employed K-fold cross validation with K = 5 on each type of data (e.g. mutations, proteomics, etc.) to assess performance. Within each K, we used leave-one-out cross-validation for each combination of data to select the alpha parameter that minimized cross-validation error. The model performance scores in Fig. 2B and Additional file 1: Table S2 represented the average correlation between predicted and actual values across all 5 models for each drug/data type. All of our analysis can be found in the `amlresistancenetworks` package we built at http://github.com/PNNL-CompBio/amlresistancenetworks and implemented at https://github.com/PNNL-CompBio/beatamlpilotproteomics. Those models that failed to select any molecular features were not included in our final analysis. The results are depicted in Figs. 2B and Additional file 1: Table S2.

### Signature interpretation using pathway annotations and statistical enrichment

To identify patterns in the features selected by the LASSO, Elastic Net, and logistic models we employed three main approaches. For gene, transcript, and proteomic signatures, we first used the `clusterProfiler` package [43] to identify GO biological process tools that are enriched for the specific genes, transcripts, or proteins selected by the model. The results are listed in Additional file 1: Table S2. In cases where there were no significant (corrected p < 0.01) terms, the column is blank. Of the 237 signatures for which the mean correlation was > 0.1, 101 exhibited some enriched terms. For phosphoproteomic features, we used the `leapR` R package [44] to identify specific kinases that were over-represented among the selected substrates, though none were identified with statistical significance. We believe this is due to the sparsity constraints imposed by the regression method as well as the large number of phosphosites for which no kinase was known, shown in Additional file 2: Figure S2B.

### Supplementing sparse regression signatures with interaction networks

To provide further context for the phosphoproteomic features selected by the models, we mapped selected transcripts, proteins, or phosphosites to published protein–RNA [45], protein-protein [46] and kinase-substrate [47, 48] interactions and then reduced this network to identify subnetworks using the Prize Collecting Steiner Forest (PCSF) R package [49, 50]. Specifically, we used the STRING database [46] together with networkKin [47] and PhosphoSitePlus [48] predictions of kinase substrate interactions to build a graph that combined protein–protein interactions with kinase-substrate interactions. To do this we added each phosphosite as its own node in the underlying graph. We weighted each edge from the node representing the substrate gene to the phosphosite with a cost of *m*/4 where *m* represents the mean cost of all the edges in the graph. The weight of each edge between the phosphosite node and the kinase gene was weighted with a cost of 3* m*/2 where *m* represents the mean cost of all edges in the graph. We then ran the PCSF algorithm [49, 50] over 100 randomizations using phosphosites, proteins, or genes from a single drug model. The results for the quizartinib proteomic and trametinib genomic logistic signatures are in Fig. 3B, D.

Using the proteins selected by the PCSF algorithm, which are a combination of those selected by the linear model as well as those selected by the PCSF algorithm, we used Cytoscape [51] and the BinGO [52] application to identify which GO biological process terms were enriched. The results are depicted in Additional file 1: Tables S3 and S4.

### Trametinib resistant cell line cultures

Human MOLM13 cells with FLT3-ITD mutation, were obtained from the Sanger Institute Cancer Cell Line Panel. Cell lines were maintained in RPMI 1640 (Gibco) supplemented with 20% Fetal Bovine Serum (HyClone), 2% l-glutamine, 1% penicillin/streptomycin (Life Technologies).Trametinib-resistant MOLM13 cell lines were generated by culturing MOLM13 cells in increasing concentrations of trametinib (Selleck). Cell viability was measured bi-weekly and cells were replenished with new media and trametinib. Resistance was assessed using the MTS assay for drug sensitivity. Once resistant, cell lines were maintained in 50 nM trametinib added bi-weekly. Cell lines were screened for mycoplasma contamination on a monthly schedule.

For proteomic and phosphorproteomic profiling, 5 million parental MOLM13 (N = 3) and resistant MOLM13 (N = 3) cell lines were starved overnight in starvation media (RPMI supplemented with 0.1% BSA). Trametinib (50 nM) was added to the starvation media of the resistant cell lines. Cells were washed three times in PBS, pelleted and flash frozen.

### Quizartinib resistant cell line cultures

Human MOLM14 cells were generously provided by Dr. Yoshinobu Matsuo (Fujisaki Cell Center, Hayashibara Biochemical Labs, Okayama, Japan). Cells were grown in RPMI (Life Technologies Inc., Carlsbad, CA) supplemented with 10% FBS (Atlanta Biologicals, Flowery Branch, GA), 2% L-glutamine, 1% penicillin/streptomycin (Life Technologies Inc.), and 0.1% amphotericin B (HyClone, South Logan, UT). Cell line authentication was performed at the OHSU DNA Services Core facility.

To establish resistant cultures, 10 million MOLM14 cells were treated with 10 nM of quizartinib (Selleck Chemicals, Houston, TX) in media alone (N = 4) or in media supplemented with 10 ng/mL of FGF2 (N = 4) or FLT3 ligand (N = 4, FL; PeproTech Inc., Rocky Hill, NJ) [53]. All cultures were maintained in 10 mL of media. Every 2 or 3 days, recombinant ligands and quizartinib were replaced and cell viability was evaluated using the Guava cell counter (Millipore Inc., Burlington, MA). Following ligand withdrawal, quizartinib and media were similarly replenished and viability was monitored every 2 to 3 days. All cell lines were tested for mycoplasma on a monthly schedule.

For proteomic and phosphoproteomic profiling, naïve MOLM14 (N = 4), quizartinib-resistant parental (N = 2, no ligand), early (N = 4/ligand) and late (N = 4/ligand) cultures were washed three times with PBS to remove any trace of fetal bovine serum, pelleted, and flash frozen.

## Results

### Multi-omic data highlights varied impact of drug response in AML patient samples

We first explored the relationship between the individual molecular (genetic, transcriptomics, proteomic, phosphoproteomics) measurements in our matched patient cohort based on known targets of specific AML drugs. Given the success of molecular profiling using RNA-seq in the Beat AML dataset [3], and general knowledge that mRNA can often, but not always, be a proxy for protein expression, we wanted to ask if mRNA and protein levels are correlated in our specific dataset. The results, shown in Additional file 2: Figure S2C confirm previously published work [9] that mRNA and protein levels are weakly correlated (Spearman R = 0.25) across all patient samples. We also mapped phosphosites to their corresponding proteins and found that the overall abundance values were also weakly correlated (Spearman R = 0.15, Additional file 2: Figure S2D), aligning with our previous work [54].

**Fig. 1:**
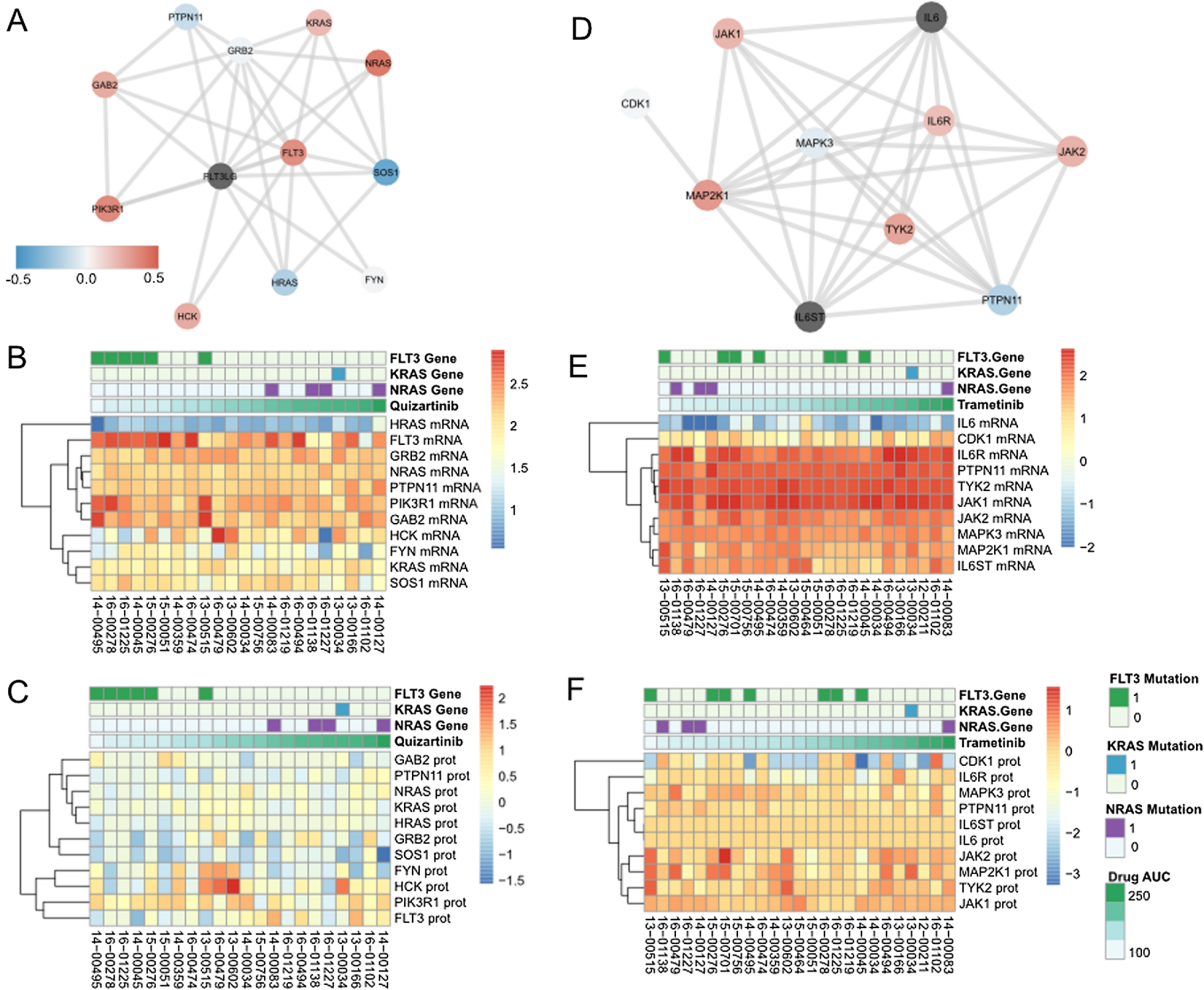
Measuring correlation across data modalities. **A** Correlation between mRNA and protein levels for individual genes in the FLT3-MAPK signaling pathway. Correlation values map to legend inset. **B** Expression of transcripts in the FLT3-MAPK signaling pathway ordered by patient response to quizartinib. **C** Expression of proteins in the FLT3-MAPK signaling pathway ordered by patient response to quizartinib. **D** Correlation between mRNA and protein levels for individual genes in the MAPK signaling pathway. Legend is the same as A. **E** Expression of transcripts in the MEK1/2 signaling pathway ordered by patient response to trametinib. **F** Expression of proteins in the MEK1/2 signaling pathway ordered by patient response to trametinib

To examine the correlation of molecular values with drug response, we first sampled sensitivity to quizartinib and the genes, transcripts, and proteins within the pathway quizartinib was designed to target. Specifically, we looked at these molecules in the FLT3/MAPK pathway (Fig. 1A) and compared them with the ex vivo sensitivity to quizartinib (Fig. 1B, C). The proteins and transcripts in the pathway itself are variably correlated. Specifically, we found that some proteins, e.g. NRAS and FLT3, are positively correlated with the mRNA levels for the same gene (R = 0.41, R = 0.34, respectively), while proteins such as SOS1 and PTPN11 are more negatively correlated (R = − 0.22, R = − 0.11 respectively). We then compared transcript (Fig. 1B) and protein (Fig. 1C) levels with the AUC for quizartinib by plotting a heatmap of the molecular values ranked by drug response. Lastly, we evaluated the phosphosites identified in our untargeted phosphoproteomics on the 12 proteins in the FLT3 signling pathway in Fig. 1A, also depicted in Additional file 2: Figure S3A. While we were unable to detect any phosphorylation sites on FLT3 itself (most likely due to the undersampling of pTyr in our workflow) we were able to characterize many alterations downstream. However, in some cases, the phosphoproteomic data will correlate with global protein levels (e.g. HCK protein expression correlated with phosphosite occupancy, with a R = 0.62, shown in Additional file 2: Figure S3B). These results suggest that protein abundance can sometimes be an effective surrogate for protein phosphorylation. The results also suggest that focusing on a single specific pathway may not be sufficient, as off-target effects of the drug that can effect sensitivity may be missed, such that integration of data could provide more meaningful results.

We expanded our correlative analysis to study the Ras/MEK pathway, which is downstream of Ras and targeted by trametinib. The correlation of the mRNA and protein levels of the pathway trametinib targets (Fig. 1D) was again modestly positive in some genes such as JAK1, JAK2, and MAP2K1 but negative in others such as PTPN11. We also measured the correlation of mRNA levels (Fig. 1E**)** and protein levels (Fig. 1F) with trametinib response in the patients. Here we found that the three patients with NRAS mutations were sensitive to trametinib, but that few other mRNA or protein levels seemed to correlate with drug response. We also studied the phosphorylation patterns of Ras/MEK targets in Additional file 2: Figure S3C, where we also found limited representation from the specific phosphosites measured. Interestingly we found examples in which phosphosite activity did not correlate with protein expression, such as MAPK3 (R = − 0.2, Additional file 2: Figure S3D). In summary we conclude that a broad, data-driven approach to studying drug response would be more successful than a targeted pathway driven approach, given the data at hand.

### Linear modeling enables broad sweep of data space to identify multi-omic signatures of drug response

Our findings in Fig. 1 show that molecular measurements across a pathway targeted by a specific drug may fail to adequately summarize the drug response in patient samples. As such, we turned to a basic statistical approach to identify such groups of genes, transcripts, proteins, or phosphosites that predict drug response.

**Fig. 2:**
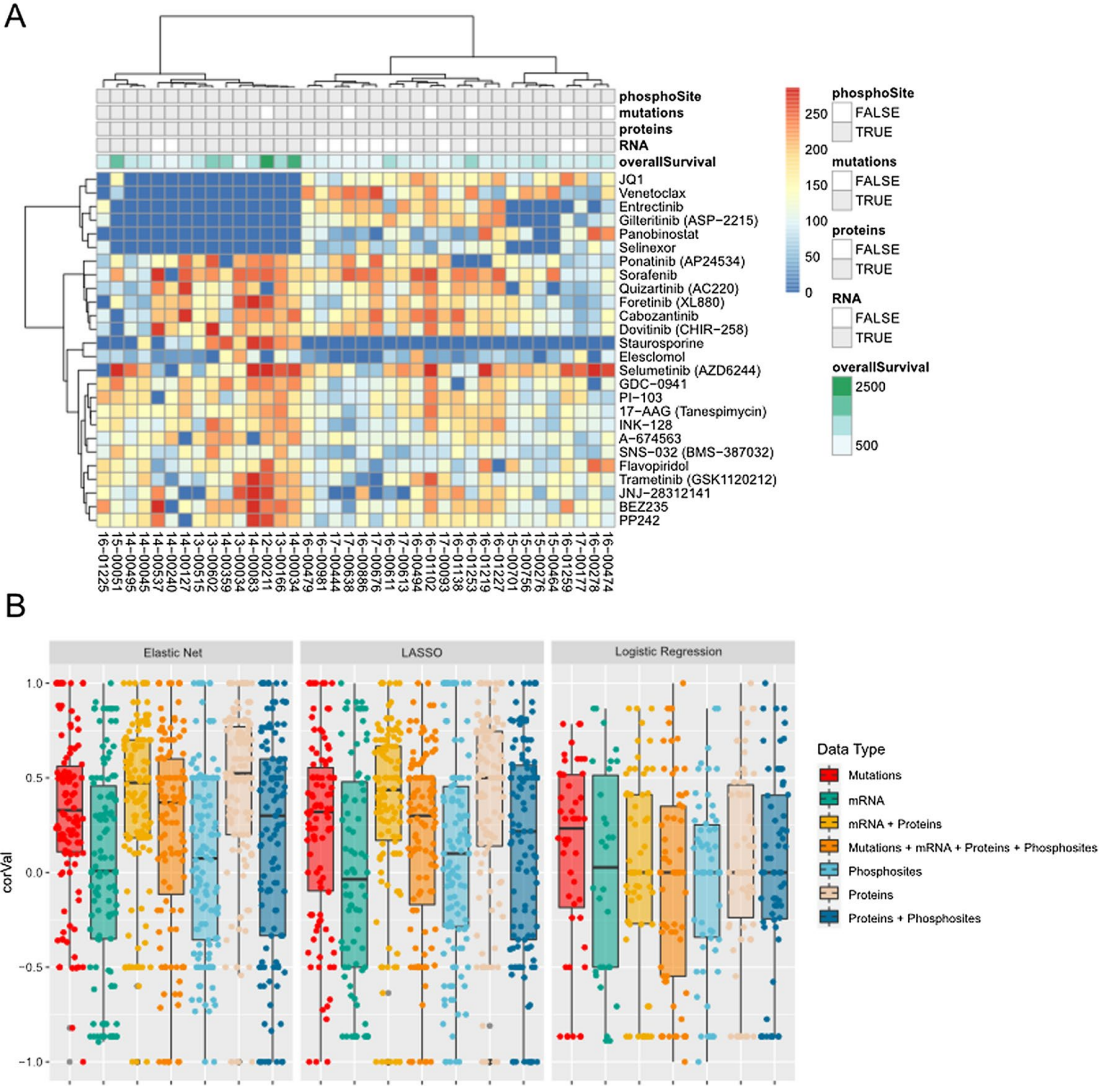
Linear modeling description and performance. **A** Summary of drug response values across 26 drugs and 31 patient samples together with the data available for each patient sample (across top). **B** Cross-validation performance of Elastic Net, LASSO, and Logistic regression with various types of data or combinations of data types. Performance is measured by Spearman correlation with held out dataset

We examined a panel of 26 drugs measured in the Beat AML ex vivo drug sensitivity functional assay described above, specifically selecting drugs that exhibited a variable response in the pilot samples as described in the experimental procedures and shown in Fig. 2A. We constructed three types of linear models as described above for each drug and data modality individually (genomics, transcriptomics, proteomics, phosphosites) as well as in combination (transcriptomics + proteomics, proteomics + phosphosites, and all four data types combined) for a total of 21 possible models for each drug. We measured the performance of each model using fivefold cross validation and measured the correlation between the predicted response on the held-out data and the actual value. The correlation values of each of the five models is shown in Fig. 2B and summarized in Additional file 1: Table S2. In numerous cases, the models were unable to select any features and therefore were not counted. This was particularly noticeable in the case of logistic regression, where the division of test data into sensitive/resistant samples left fewer data points for model construction.

This framework enabled us to compare modeling approach and data type. While all three flavors of regression performed similarly, the logistic regression created fewer models and was not very accurate overall (median correlation < 0.1, Fig. 2B). The other two regression models showed a significant boost from adding the proteomics data to transcriptomics data (mRNA + Proteins, yellow, Fig. 2B). Interestingly, the strongest overall performance comes from proteomics data alone (beige). Despite the general good performance of models, there was a high degree of variability between drugs and drug families. Additional file 2: Fig. S4 shows the performance of each model across individual drugs (Additional file 2: Fig. S4A) and drug classes (Additional file 2: Fig. S4B). This diversity shows that individual model selection requires a robust cross validation approach to avoid generalizing with only one type of model or data modality.

### Model selection via cross-validation and network analysis provides robust interpretations of molecular signatures

**Fig. 3:**
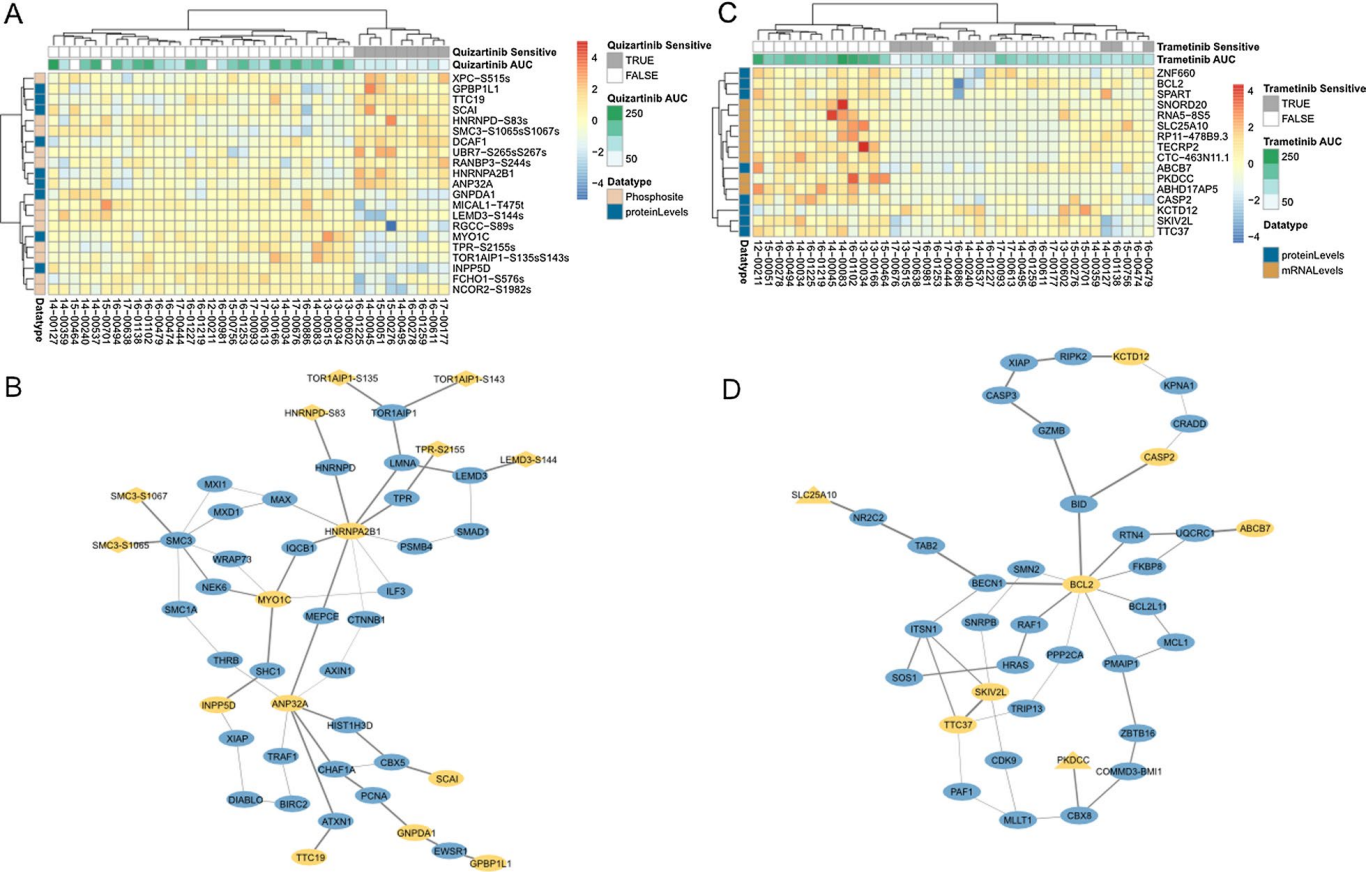
Interpretation of top-performing signatures by heatmap and protein network integration. **A** Heatmap of proteins and phosphosites selected by the logistic regression depicts clustering of patients by sensitivity to quizartinib. **B** Interaction network links proteins (ovals) and phosphosites (diamonds) selected by signature (yellow) to other proteins (blue) to illustrate how they relate to one another. **C** Heatmap of proteins and transcripts by the LASSO regression shows how they cluster patients by trametinib AUC. **D** Interaction network showing how those proteins (ovals) and transcripts (triangles) selected by the signature (yellow) are closely related via protein interactions with related proteins (blue)

To show how the cross validation framework can be used in practice, we selected the top performing models of quizartinib and trametininb response from Additional file 2: Fig. S4A and Additional file 1: Table S2 and examined the features to determine if they aligned with the known mechanism of action of each of the drugs. The top-performing (via average correlation with held-out data) models that predict quizartinib and trametinib response are depicted in Fig. 3.

We first analyzed the features from the logistic regression model of quizartinib response based on proteomic and phosphoproteomic data. To identify the features that drove the models, we re-ran it on all the data (instead of on just the training data subsets) and plotted the features in Fig. 3A. Here we noticed INPP5D, which is identified in both the LASSO and logistic regression models and highly down-regulated in quizartinib sensitive samples. This gene encodes the inositol 5-phophatase know as SHIP-1 which acts as a negative regulator of the PI3K/AKT pathway. SHIP-1 affects cell proliferation in AML, due to mutations in the nuclear localization signature or phosphorylation site [55]. It has also been shown to act as an adaptor protein linked to wild type FLT3 signaling [56, 57].

While we found two enriched GO Biological process terms related to actin filament-based transport, (Additional file 1: Table S2) in the regression signature, we employed a network approach to better characterize how the selected phosphosites and proteins interacted based on published protein–protein and kinase-substrate interactions. We used the OmicsIntegrator [49] tool to supplement the proteins and phosphosites selected by the model (yellow nodes in Fig. 3B) with proteins from the protein protein interaction network (blue nodes in Fig. 3B). This approach enables improved visualization of protein and phosphosite activity by linking together individual signature components. For example, SHC1 was added by the network algorithm, which has been found to be expressed in AML blasts [58], as well as highlighting the role of phosphorylation of SMC3, a member of the cohesion complex, which has been also found to synergize with FLT3 in AML [59]. By linking the proteins in the signature together through other protein interactions, we can examine how the proteomic signature connects various proteins involved in signaling, histone regulation, and DNA damage to show how alterations in diverse pathways give rise to drug sensitivity (Additional file 1: Table S3).

We also examined the top model that predicted trametinib response from Additional file 1: Table S2, which is comprised of both transcripts and proteins (Fig. 3C). In this case, expression of the signature proteins and transcripts was able to cluster highly resistant patient samples on the left of Fig. 3C. Biological process enrichment (Additional file 1: Table S2) included terms related to mRNA processing and catabolism. When we used the mRNA and proteins to build a network using the PCSF algorithm, depicted in Fig. 3D, we identified numerous additional apoptotic related proteins, such as BID, CASP1, and GZMB that suggest that expression of apotosis-related proteins and transcripts could predispose patients to trametinib sensitivity. The proteins in the network were broadly enriched in apoptotic related pathways (Additional file 1: Table S4), suggesting that this pathway plays a role in MEK inhibitor response. This hypothesis has been confirmed by the apparent synergy between venetoclax, a BCL2 targeting drug, and other MEK inhibitors used for treatment in AML [60].

### Proteins that predict drug response are dysregulated in resistant cell lines

**Fig. 4:**
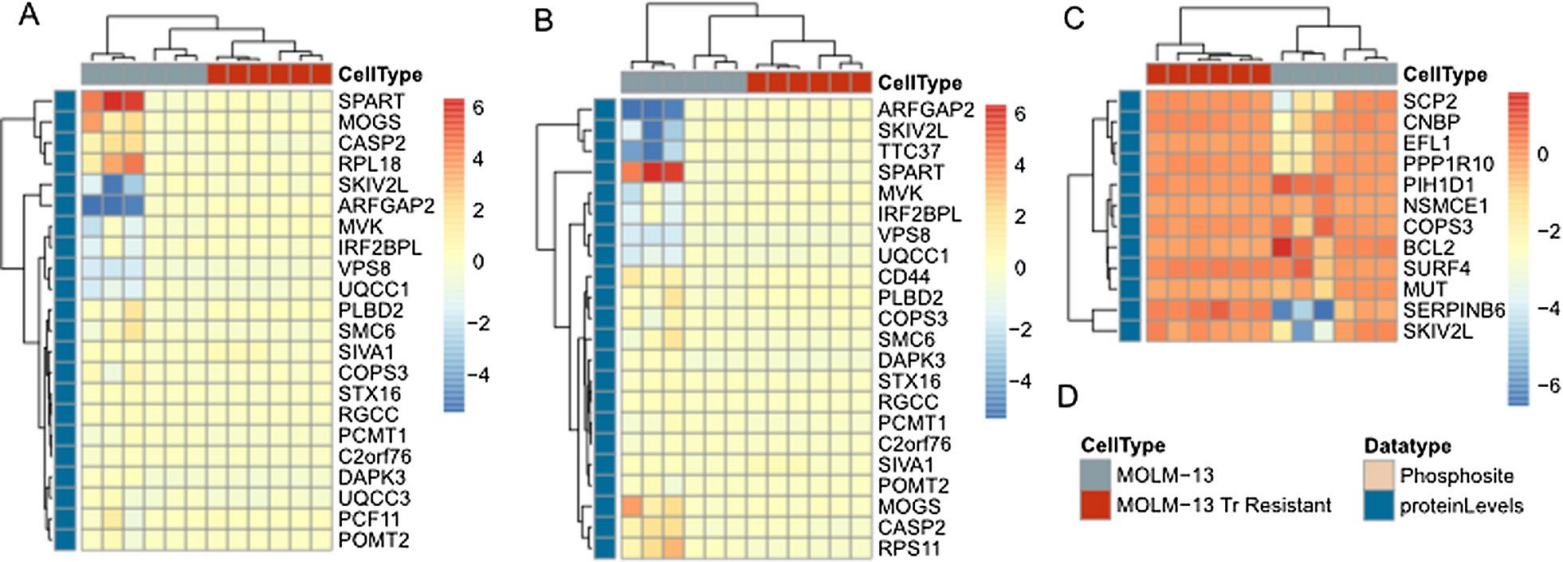
All proteomic signatures selected from ex vivo modeling cluster MOLM13 parental cell lines from those that are resistant to trametinib. **A** Heatmap of signature selected from LASSO regression. **B** Heatmap of signature selected from ElasticNet. **C** Heatmap of signature selected from logistic regression. **D** Legend

To experimentally validate these signatures, we turned to cell culture models of AML. Here we explicitly focused on proteomic and/or phosphoproteomic measurements to determine if protein and phosphosite levels could predict resistance to drugs in vitro*.* We first examined MOLM13 cells that were grown in the presence of trametinib over 3–4 months to develop resistance, and measured protein expression in the resistant cells compared to the parental cells. While the regression modeling selected a combination of transcripts and proteins to be the most informative (Additional file 1: Table S2 and Additional file 2: Fig. S4A), we only had protein measurements from these cell lines, and therefore clustered the proteins from the proteomic signatures of trametinib response in these cell lines (Fig. 4). Despite the fact that the proteomic signature was not as robust in our cross-validation compared to that of proteins together with mRNA, we found that each protein signature from the LASSO, logistic, and Elastic Net regressions cleanly clustered resistant and sensitive cells. This suggests that the proteins derived from these signatures represent the biological indicators of trametinib response.

### Proteomic signatures can distinguish between early and late models of drug resistance

**Fig. 5:**
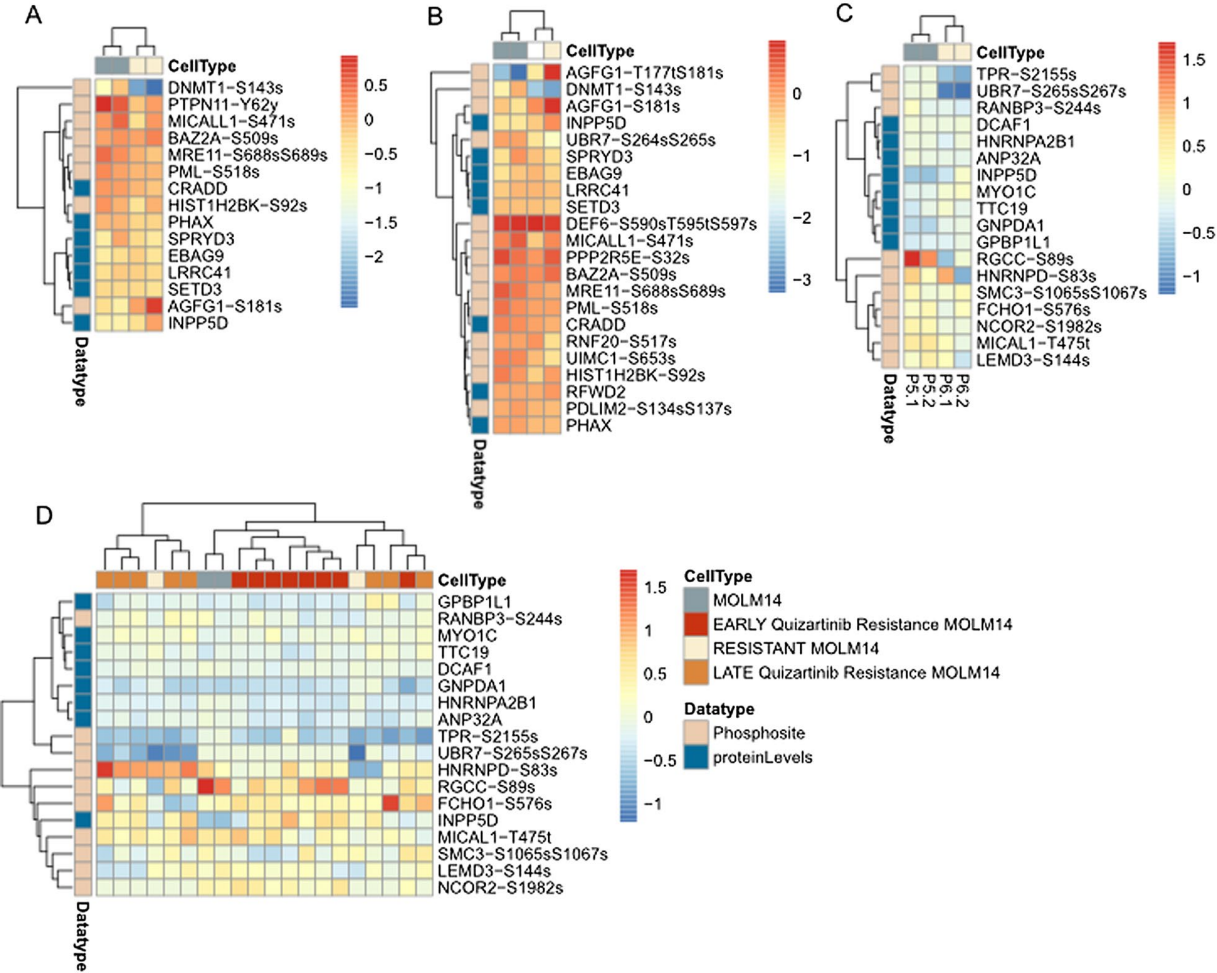
Proteomic signatures selected to predict ex vivo response to quizartinib using the **A** LASSO, **B** Elastic Net, and **C** logistic regression cluster quizartinib resistant cells from parentals. **D** The same signature from (**C**) but with additional cell lines that were developed as models of late and early resistance

To further confirm this role of regression-derived signatures in cell lines, we evaluated the proteins and phosphosites selected for the quizartinib signatures in MOLM14 cells grown in the presence of quizartinib to develop resistance. We then examined the proteins/phosphosites selected by all three regression models and found that regardless of which method, the proteins clustered the resistant and parent cells separately (Fig. 5A–C).

We then wanted to explore if any of these signatures were related to temporal changes during development of resistance to quizartinib. We used resistant cell lines that were developed in two stages: early resistance, which is mediated by extrinsic ligands from the marrow microenvironment, and late resistance, which is mediated by the expansion of intrinsic resistance mutations—most commonly in the activation loop [53, 61]. Using this model we compared early resistance and late resistance, with the hypothesis that the patient-derived signature would more closely resemble the late resistance phenotype.

To test this hypothesis, we plotted the proteins and phosphosites selected by the logistic model (which was the best performing according to Additional file 1: Table S2 and Additional file 2: Figure S4A) in these cell lines and clustered them in Fig. 5D. We observed a similar split between sensitive and resistant cells as we did in Fig. 5A–C, as the proteins that predict drug response cluster the MOLM14 parental cells (blue) distinctly from the fully resistant cells (beige). However, in this case, these proteins also separate those cells that represented early resistance (red) from those that represent late resistance (orange) in our previous work. This fits with our previous claim that the resistance to FLT3 inhibitors involves a two-step process, as cell lines exhibiting the early resistance phenotype cluster more closely with the parental cells than with the late resistance cells.

## Discussion

This study describes a computational framework to asesss the role of protein-derived measurements in predicting ex vivo AML patient drug response. Given that proteins and phosphosites clearly capture a unique aspect in disease activity, we employed numerous types of regression analyses together with cross-validation to determine the best signature for each drug or drug family. We also showed how to interpret these signatures using data from external sources and validated the signatures in cell culture models.

This study was not without limitations. For example, this was a small dataset, with only 38 patients, so is limited in the diversity of drug response as well as mutational heterogeneity. Furthermore, the depth of coverage of phosphotyrosine sites was limited in our untargeted phosphoproteomics is (Additional file 2: Fig. S2B), which can be particularly challenging when trying to evaluate the impact of drugs targeting FLT3, a kinase that primarily operates through tyrosine phosphorylation. As such, we illustrate in Fig. 1 that evaluating signaling pathways targeted by each drug may not be sufficient across high throughput datasets, so rather used linear models to identify specific features that are better characterized in the available data we collect. We are working toward both increasing our sample size in future studies as well as sorting cells to identify signatures (from single cell transcriptomics or proteomics data) across various cell populations within AML patient samples to improve our ability to predict drug response.

While we were able to compare different flavors of regression modeling, we believe that, for our data, there is no best choice across all drugs. The logistic regression failed in many cases with low sample numbers, so may not work for all drugs. However, the choice of data type does seem to have more of an impact, as genetic mutations are robust in cases of targeted therapy (e.g. trametinib and quizartinib for NRAS and FLT3 activating mutations respectively), but models involving proteins perform best when assessed over all drugs (Fig. 2B and Additional file 2: fig. S4). We are looking to expand this analysis using a larger patient cohort where we can further derive robust protein signatures that can be validated in the clinic. We believe that our protein-based approach can be easily employed in the clinic through antibody-based measurements targeting a small set of proteins that can more rapidly predict drug response than current genetic-based assays.

We also underscore the need for interpretable models for drug response. While the regression approaches select the features that are numerically most valuable for predicting drug response, they fail to account for the biological context of the proteins or genes selected. As such, we believe that using the OmicsIntegrator or other tools to map selected proteins to the interaction network will provide better understanding of what causes drug resistance in some patients, and potentially assist in understanding the effects of drug combinations, which are becoming increasingly common in clinical trials [62], 62].

In summary, this study presents an effective workflow for the future analysis of integrated genomic, transcriptomic, proteomic and phosphoproteomic data in larger cohorts, such as the larger Beat AML cohort (N = 210). While the patient cohort used in this preliminary study is limited in size, the robust verification of results in independent cell line studies provides confidence in the scalability of these methods. Additionally, the performance of protein-based models compared to transcriptomic-based models opens up the possibility of developing antibody-based, CLIA-eligible assays for the rapid assessment of likely therapeutic targets at the time of biopsy, without the need for DNA or RNA sequencing. Lastly we believe that our network approaches could help identify potential novel drug synergies that could be tested in the clinic.

## Supplementary Information

**Additional file1: Table S1**: List of drugs and available samples. **Table S2**: All identified signatures and their functional enrichment (GSEA/KSEA). **Table S3**: GO enrichment of network in Figure 3B. **Table S4**: GO enrichment of network in Figure 3D.

**Additional file2: Figure S1:** Overview figure describing the experimental design. **Figure S2**: Counts of distinct data types, and correlations between them. **Figure S3**: Examination of phosphosite measurements in FLT3 and Ras/MEK pathways. **Figure S4:** Summary of model performance by drug and drug family.

## Data Availability

Data was uploaded to Synapse where it was used for subsequent analysis at http://synapse.org/ptrc. mRNA (counts per million) and genetic mutation measurements (variant allele frequency) can be found at https://www.synapse.org/#!Synapse:syn22172602/tables/. All data used for this project is stored on Synapse at http://synapse.org/ptrc, where you can request access to the data specifically mentioned in this manuscript. All analysis and figures can be viewed at https://github.com/PNNL-CompBio/beatamlpilotproteomics.

## Abbreviations

AML: Acute myeloid leukemia
MS: Mass spectrometry
CPTAC: Clinical proteomic tumor analysis consortium
FDR: False discovery rate
AUC: Area under the curve
