# Supplementary material for: Proteomic and phosphoproteomic measurements enhance ability to predict ex vivo drug response in AML
Source: Clin Proteomics. 2022 Jul 27;19:30. doi: 10.1186/s12014-022-09367-9 (PMC9327422; doi:10.1186/s12014-022-09367-9)
Supplement: Supplementary file 2 — Additional file2: Figure S1: Overview figure describing the experimental design. Figure S2: Counts of distinct data types, and correlations between them. Figure S3: Examination of phosphosite measurements in FLT3 and Ras/MEK pathways. Figure S4: Summary of model performance by drug and drug family. [file 12014_2022_9367_MOESM2_ESM.docx]

Figure S1: Overview figure describing the experimental design.


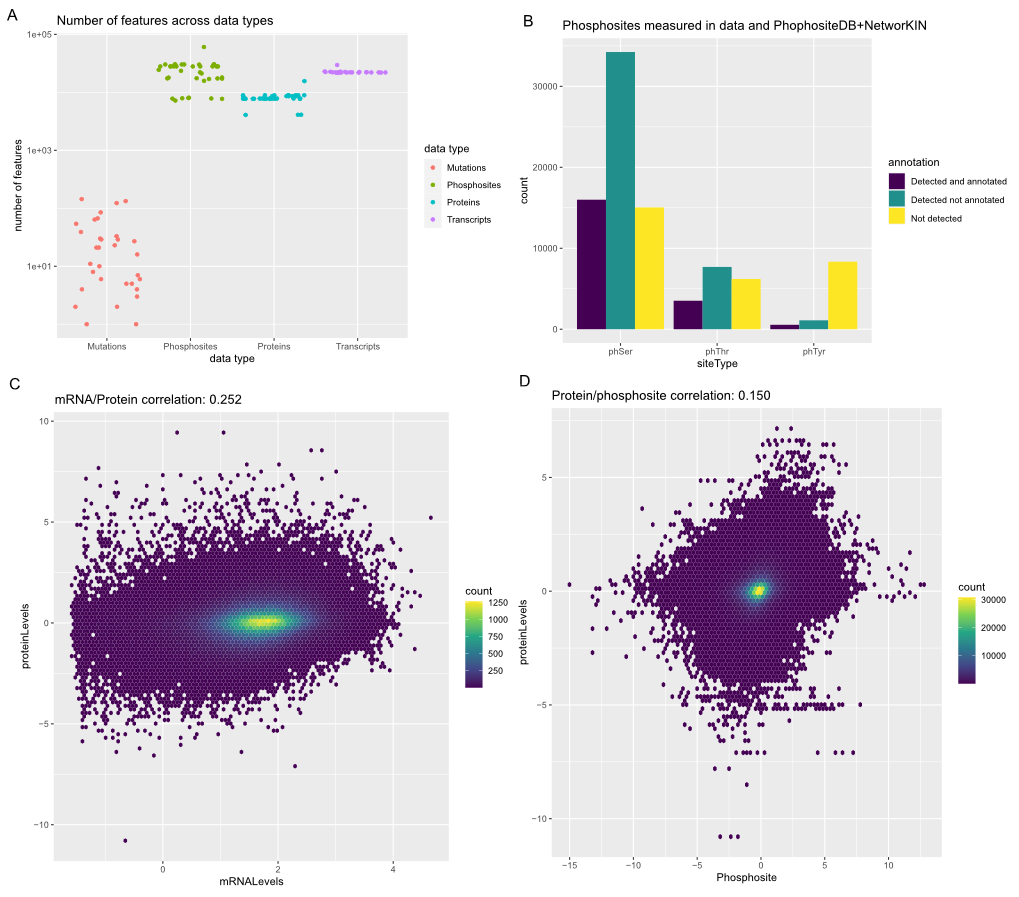
**Figure S2**: Individual measurements of mRNA, mutations, protein, and phosphosite alongside correlation analysis of mRNA, protein, and phosphosite across all patient samples. (A) Number of individual features used for modeling across al patients. (B) Number of phosphosites detected across the study that were both annotated (purple) and not annotated (turquoise) in PhosphositeDB and NetworKIN. Yellow bar indicates sites in database that were not measured. (C) correlation of mRNA and proteins across samples. (D) Correlation of protein and phosphosites across samples.


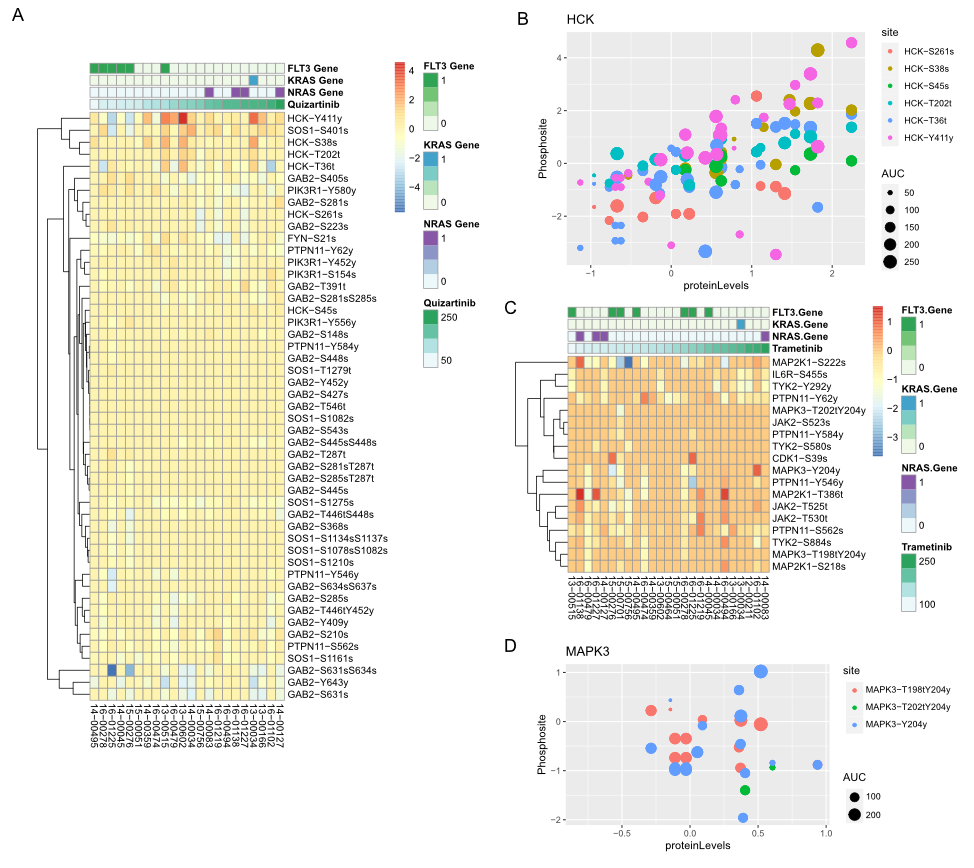
**Figure S3:** Role of phosphorylation in Quizartinib and Trametinib-affecting pathways.(A) Expression of phosphosites in proteins in the FLT3 signaling pathway that were detected in our dataset. Quizartinib sensitivity (AUC) and genetic mutation status. (B) Example of correlation between phosphosite expression and protein expression in HCK. (C) Expression of phosphosites in proteins in the MEK signalign pathway. That were detected in our dataset, together with sensitivity to Trametinib and mutational status. (D) Expression of MAPK3 protein shows inverse correlation with some phosphosites on MAPK3.


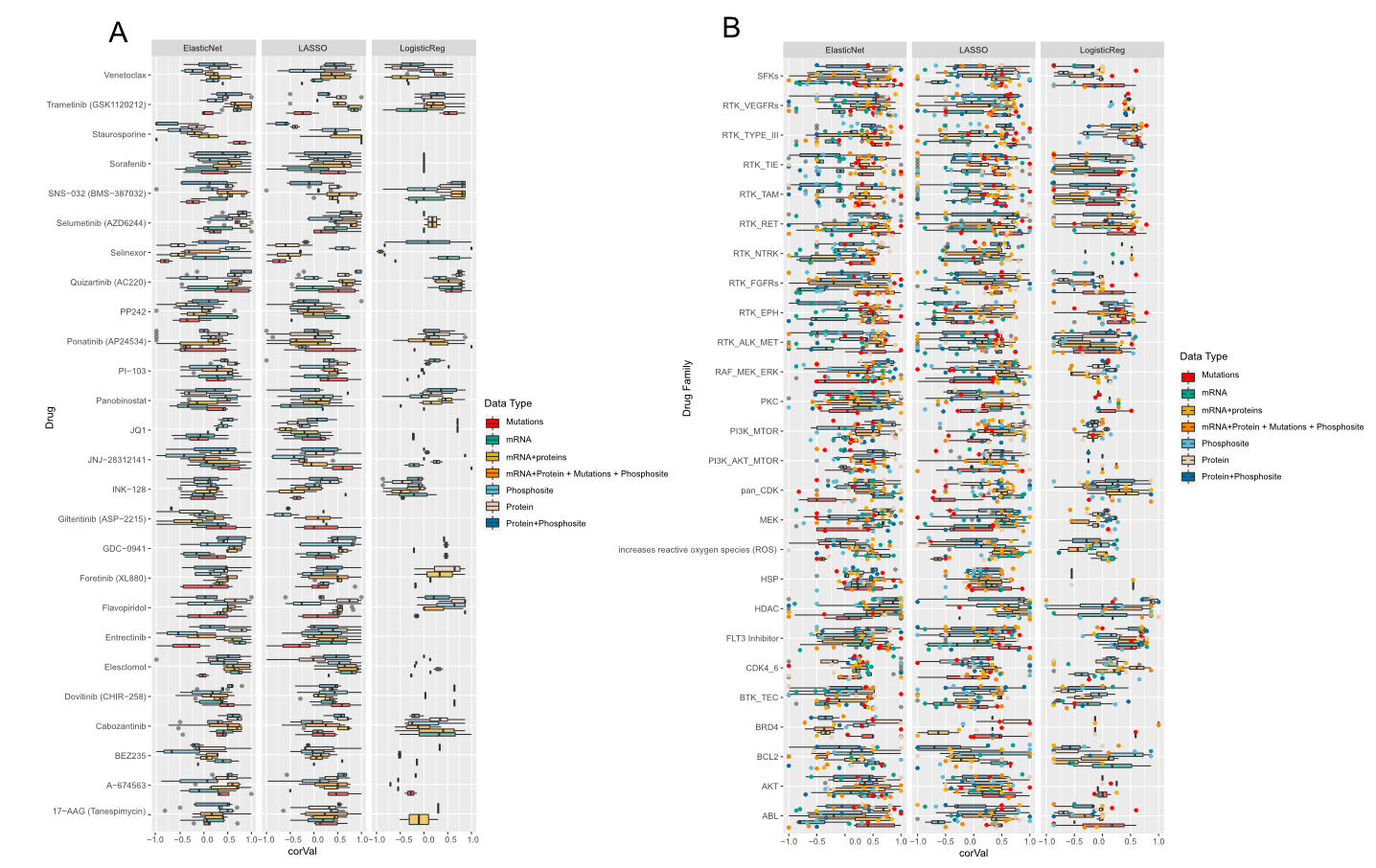
 **Figure S4**: Correlation of held-out test data with predicted values across various model and data combinations. (A) Spearman rank correlation of predicted AUC of drug response across individual drugs measured using a Logistic regression, LASSO regression, and Elastic Net regression. We evaluated each model using mutation data (red), mRNA levels, (green), protein levels (blue), phosphosite measuremetns (orange) or all data elements combined (yellow). (A) Performance of each of the cross-validated models separated out by drug. (B) Performance of each of the models trained by drug family.
